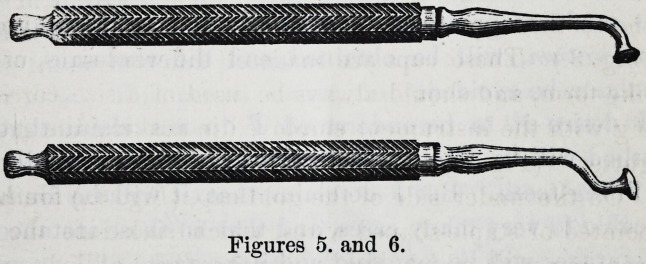# On Filling Teeth

**Published:** 1876-03

**Authors:** Geo. H. Perine

**Affiliations:** New York.


### ARTICLE IV.

### *On Filling Teeth.*

BY GEO. H. PERINE, D, D. S., NEW YORK.

In considering and investigating subjects within the province of the student or practitioner in dental science, there will perhaps, be found none other which possesses to the same degree, the general importance and magnitude as an interest which characterize that of *filling teeth.*

And this is because of the constant presentation in practice of cases in this branch of dentistry, and therefore of the wide spread range of dental surgery precisely in this direction; and while among many, the mere mechanical operation of filling teeth, is considered as one of the lesser branches of the science, this theory is at once flatly contradicted by the fact that no other topic in dental surgery has received so much or so exhaustive treatment in the way of writing and of oral teaching as well.

And yet although so much has been said upon the subject, and so well, I do not believe the topic is by any means exhausted. In fact, while humanity exists, and there thus remain teeth to be filled, if we may be guided by experience in the past in all science, we may properly believe that improvements in practice will constantly occur, that new and valuable and pertinent inventions will be made, and that our general knowledge of the subject in all its ramifications will grow with our growth.

It may not therefore be considered improper for me to endeavor in my turn to add some little to the aggregate wisdom of our profession in this matter, and thus naturally add to the comfort and health of those whom we are educated and skilled to serve. I therefore propose to lay before your readers an improved process or method in this department of dentistry. And in so doing, I desire to say that I do not thus venture upon professional publicity, without having concentrated for a length of time my thoughts upon the subject in question, nor without having established in so far as experiment will establish anything, the accuracy and justice of my preconceived views upon this subject. Now I may premise the following proposition, to wit:

That in the practice of the profession, it will possibly be found in a very large number of fillings, in a certain class of cases, and in certain temperaments and certain patients, that even the most carefully prepared and adjusted fillings do not stand the test of time.

So much depends upon conditions having no relation to the case and precision of the operator, that to obviate the influence of these conditions when deleterious, should be the first consideration in such practice.

But precisely how to do this has been, I imagine the stumbling-block in this class of dental operations, and this is the reason why many fillings have not possessed more of a permanent character. In cases of the teeth of young persons, and always in that of teeth possessing more than the ordinary sensibility, and when their vitality is endangered, some change in the customary mode of operation seems needed.

It is generally admitted that much injury is occasioned by thermal changes operating through the employment of mental fillings, approximating to the pulp. To obviate this difficulty, many experiments have been tried, and new theories revised, but thus far in my knowledge without success.

As regard the details of such cases, I have only to remark that such teeth are too often filled with amalgam; gutta-percha and os-artificial being in my judgment, a better material to employ, provided that the masticating or other external surface can be protected from friction.

Many operators treat such cases by underlaying with plastic filling, and building up with gold foil. But in general practice the operation is protracted and tedious, thus wearing upon the nervous system of the patient, and producing results (frequently) which are in the highest degree objectionable. I propose by my method to obviate these disagreeable features.

I undertake to make an operation which shall be perfect in its result, while possessing none of the painful, annoying or injurious characteristics of the customary building up process—an operation in fact, whose quality when completed, no dentist can justly question. It should be borne in mind however, that while my method is greatly simplified, this fact should not induce any carelessness on the part of the operator, in the matter of giving due protection from the action of the fluids of the mouth, while the operation is in progress; and also, that no point in the proper preparation of the tooth for the filling should be slighted.

I may remark here that where the operator is met by a case of sensitive dentine, he may properly make use where it can be safely employed, (avoiding contact with other than the tooth to be operated upon,) the cautery battery, in preference to resorting to powerful medical applications.

The method which I offer to the profession, is used in connection with a plastic, non-conducting material, applied as heretofore observed and with proper care, and for this purpose, I would recommend Hill's stopping or ostificial.

Having proceeded in this manner, I protect the plastic stopping with a perfectly fitted cap of gold plate. In explanation of this method, I will state that in preparing the tooth for filling, I employ an instrument sufficiently large to reach the healthy part of the tooth surrounding the cavity to be filled ; with the instrument, (Fig. 1.) I form a perfectly circular cavity, taking care not to approach too near the lining pulp or nerve.

Fig. 1.

Fig. 2.

Fig. 3.

Fig. 7.

The instruments illustrated in Fig. 1, 2, may be used on either of the dental engines.

After the cavity is properly prepared for the reception of the stopping, with the second instrument, (Fig. 2 ,) I cut around the edge of the cavity on the external surface of the tooth, a groove or gutter, in which to set the gold cap. (Fig. 3.) These caps are made of different sizes, of a cup like form, and should always be used of a size corresponding with the instrument employed in making the groove or gutter. (Fig. 2.)

On the under side of the gold cap, (Fig. 3.,) are hooked, pointed or staple-shaped wires, which sink into the plastic stopping with which the cavity has been filled, and thus serve to anchor the cap ready for finishing whenever the material has hardened—as in os-artificial, or cooled as in Hill's stopping.

Figure 4.

If Hill's stopping be used, the cap must be warmed before being applied, by means of the instrument, (Fig. 4.,) by which it is first carried to its place, then with instrument (Fig. 5 and 6,) by gentle pressure the cap is pressed home, causing the border of the cap to spread into the gutter or groove. It will be found that the proper adjustment of the cap in place, depends upon the force used in pressing it home upon the plastic stopping. It will also be observed, that the pressure will force the surplus material out under the edges of the cap, and thus on hardening or cooling, will complete the union between the cap and stopping; of course the force necessary must be applied with great care and judgment. The instruments with which this part of the operation is performed is illustrated in Figs. 5 and 6, being larger than the external cavity, rests as it is pressed home, upon the surrounding edge, and thus the centre of the cap is prevented from being forced too far into the cavity, the result of which would be the lifting of the border of the cap from the groove or gutter, before the material had set.

Figures 5. and 6.

The gold caps are manufactured in various sizes, to obviate the necessity of using such as would be larger or smaller than the nature of the case required—Fig. 7 illustrates a gold cap with flange border. After it has been placed in position—a rope of gold foil may be packed in and around the border of the cap, and afterwards burnished down. Where the surface upon which the cap is fitted, is irregular, the cap and gold foil may be burnished down to fit into the inequalities, rather than use the file to remove them.

Caps made of mother pearl, may be used in the same manner as the gold caps, in such cases as may render the use of the latter offensive to the eye or otherwise objectionable.

These caps are similar in their construction to those of the gold, with the exception, that in the place of the points, hooks, or staples, by which the latter attaches firmly to the plastic stopping, we have dovetailed projections which fulfill the same object.

I claim for this new method of filling teeth with the appliances and instruments which I have described, that a more satisfactory, certain and permanent operation may be performed in certain cases, than by the ordinary method at present adopted.

I am of the opinion that the profession will meet with a large number of cases in their practice, in which this method may be adopted, with advantage to themselves and to their patients. And furthermore, I believe that a greater number of teeth will be saved, and for a longer period by this mode of practice. And finally, that in this way great *diesderatum*, will be obtained, and the wholesale use of amalgam be avoided.

I desire it to be understood I do not claim that this method will be found practicable or desirable in all cases of filling teeth. But I do claim that it will be found applicable to very many cases, and that in those instances its advantages will be manifold and important.

## Figures and Tables

**Fig. 1. f1:**
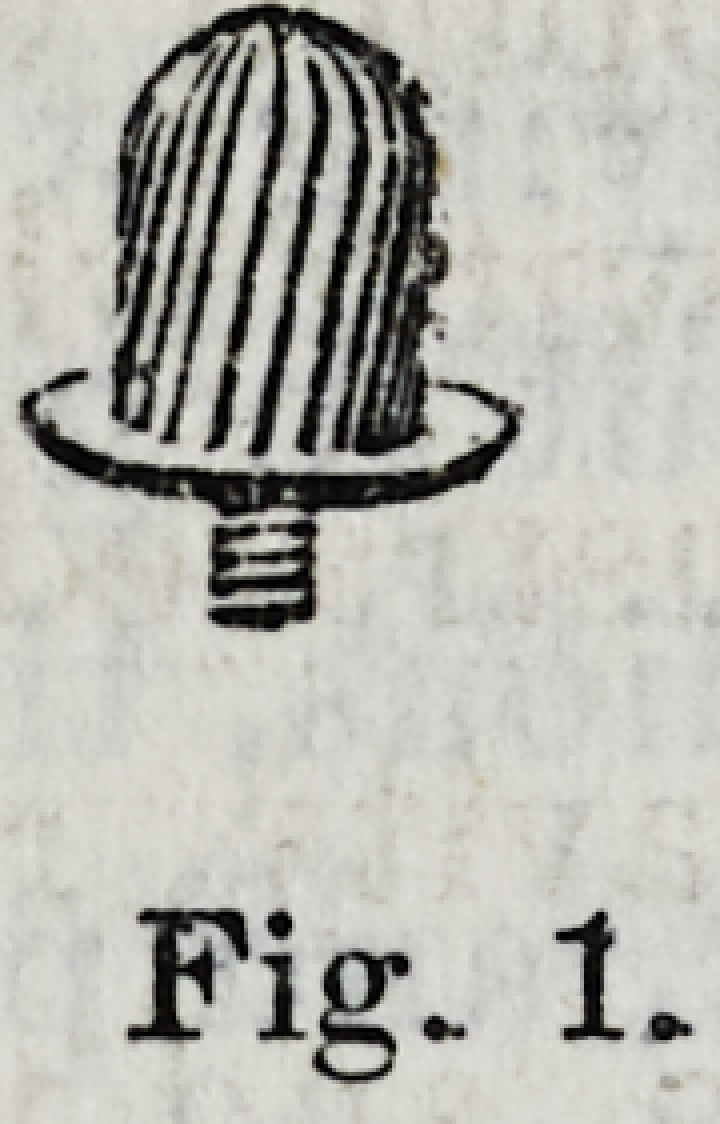


**Fig. 2. f2:**
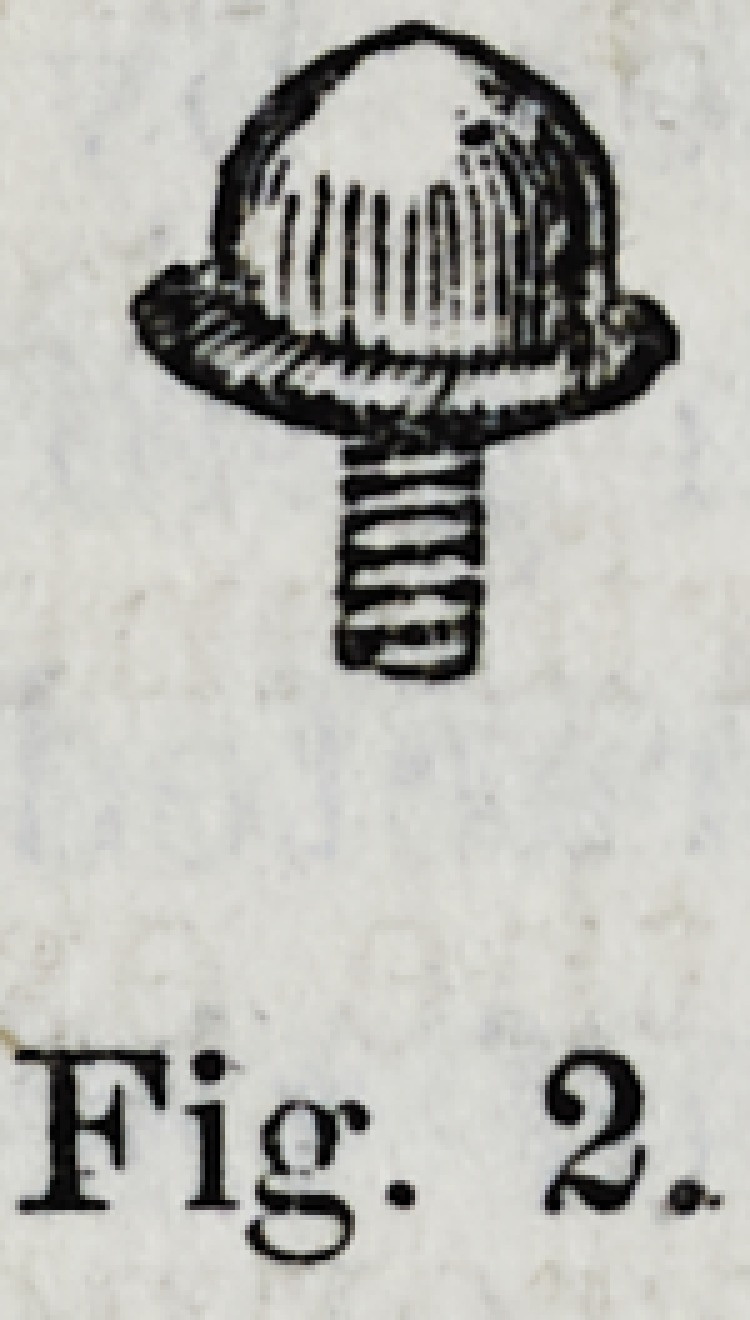


**Fig. 3. f3:**
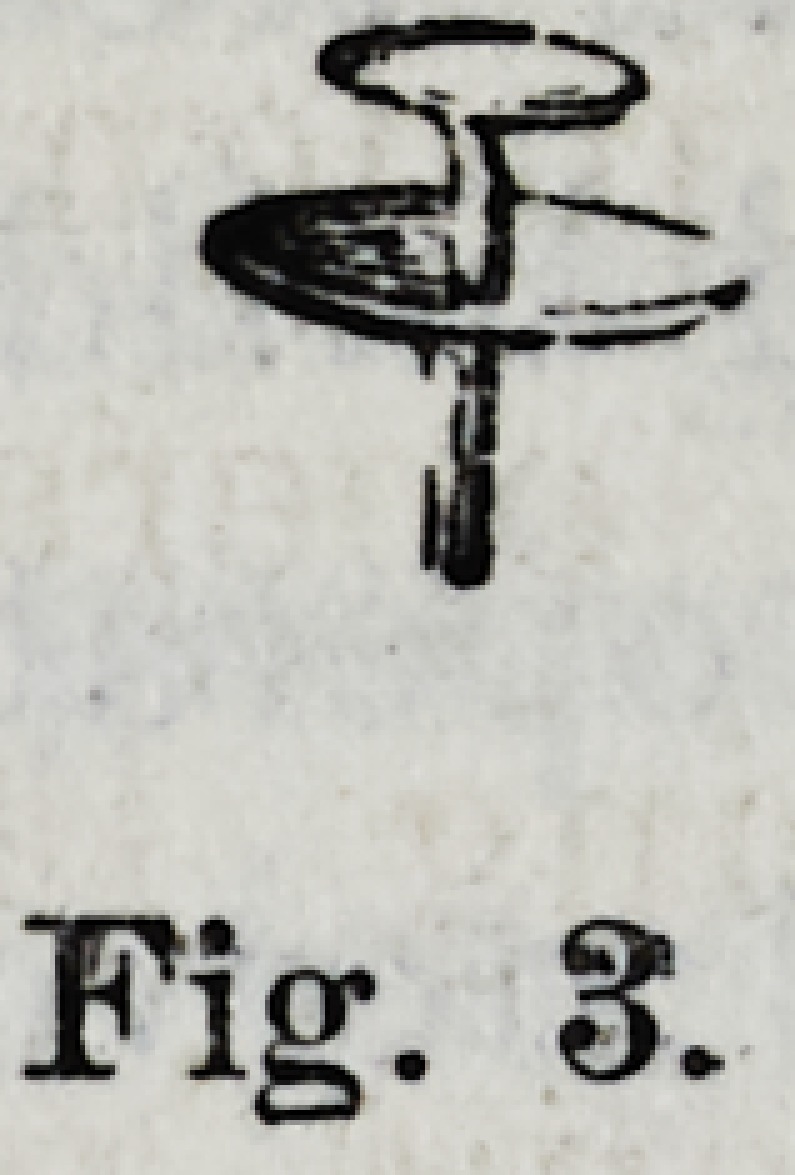


**Fig. 7. f4:**
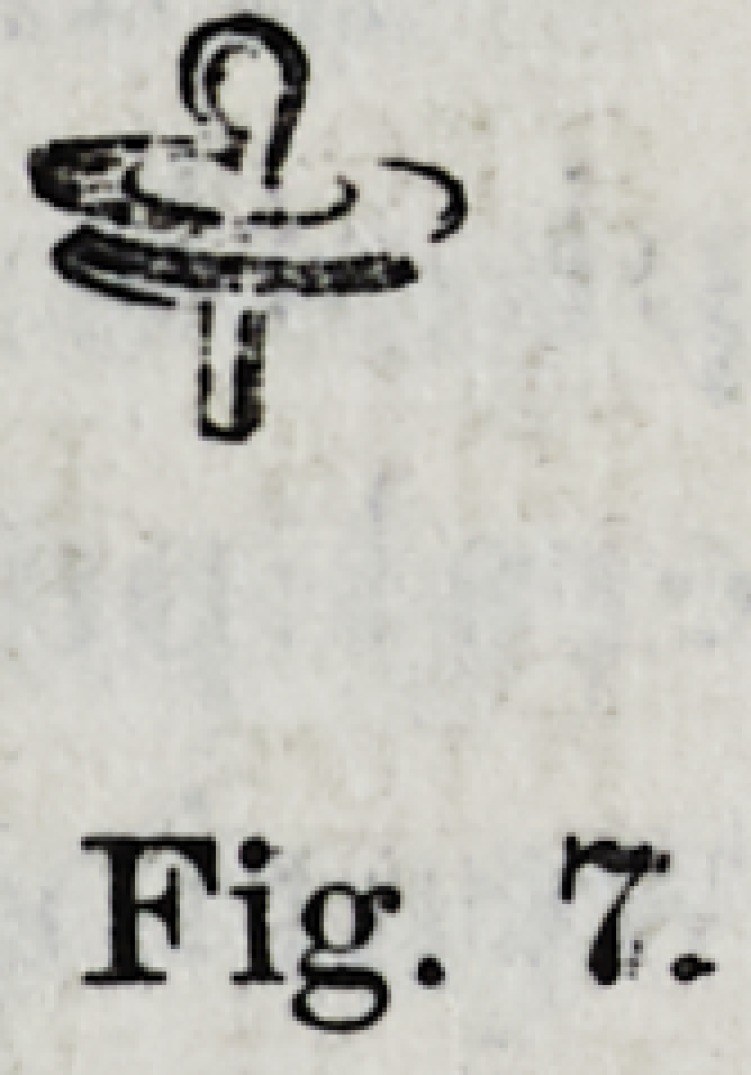


**Figure 4. f5:**
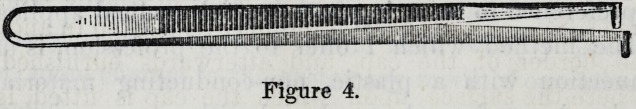


**Figures 5. and 6. f6:**